# Temporal dynamics and analysis of pollutant parameters in the water quality entering a reservoir dam

**DOI:** 10.1038/s41598-026-42878-1

**Published:** 2026-03-25

**Authors:** Raoof Mostafazadeh, Tayebeh Irani, Saeid Mousavi Moghanjoghi, Bita Moezzipour

**Affiliations:** 1 Department of Natural Resources and member of Water Management Research Center, Faculty of Agriculture and Natural Resources, University of Mohaghegh Ardabili, Ardabil, Iran; 2 Watershed Management Science and Engineering, Department of Range and Watershed Management, Faculty of Natural Resources, Urmia University, Urmia, Iran; 3 Department of Environmental Sciences, Islamic Azad University Damavand, Tehran, Iran; 4 Department of Natural Resources, Faculty of Agriculture and Natural Resources, University of Mohaghegh Ardabili, Ardabil, Iran

**Keywords:** Water Quality Parameters, Pollution Monitoring, Water quality dynamics, Pollution control, Graphical analysis, Environmental sciences, Hydrology, Chemistry

## Abstract

Assessing water quality changes across water bodies is essential for resource management and environmental protection, as variations influenced by natural and human factors help predict trends and control pollution. This study evaluates temporal trends in key physical, chemical, and biological water quality parameters from 2014 to 2024, entering the Sardasht Reservoir Dam, located in the mountainous region, in order to assess the effects of pollution, natural changes, and human activities on water quality. The data includes physical parameters (temperature, EC, TDS), chemical parameters (BOD, COD, nitrates, heavy metals like Mn and Pb), and biological parameters (total coliforms). The research applied statistical methods, including the Mann-Kendall test and Sen’s slope, to determine trends in these parameters over time. In addition to trend analysis, the study utilized box plots, violin plots, and probability density functions (PDFs) to understand the distribution and variability of water quality values. The results reveal several important trends in water quality at Sardasht Dam. COD showed a significant increasing trend (Tau = 0.224, *p* = 0.033), indicating rising organic pollution. Several parameters, including TDS and pH, exhibited decreasing trends, though not statistically significant. Mn levels and Temp showed increasing trends, possibly linked to environmental changes like industrial activities or climate change. Significant fluctuations in COD, NO3, and Pb suggest episodic pollution events, potentially from urban or agricultural runoff. DO, pH, and TDS displayed more consistent trends, showing stable water quality in these aspects. These results indicate the rising COD and temperature posing potential threats to water usability and ecosystem health. Elevated manganese levels may lead to sediment accumulation and water treatment challenges. The study recommends closer monitoring and management of inflows to the reservoir, particularly addressing organic pollution, temperature increases, and metal contamination to maintain water quality standards. The results emphasiz the need for strategic pollution control measures, further emphasizing the importance of long-term monitoring in this key water resource. The results show the urgent need for integrated pollution control and sustainable water management strategies to maintain water quality and support long-term reservoir operation.

## Introduction

### Background

Over time, with the expansion of human societies and increased water use, water quality has deteriorated, with over 80% of wastewater likely released untreated into the environment, making surface water pollution a global issue^1–4^. As water resource development plans primarily aim to ensure reliable and high-quality water for various uses, assessing water pollution is crucial^5^. Today, environmental laws and issues related to water pollution and quality make it essential to focus on the dynamics of water quality parameters^6^. Population growth and pollution from urban, industrial, and agricultural waste, landfill leachates, and surface runoff have expanded pollution and limited water resources^7–10^. Given the growing pollution sources, surface water bodies such as lakes, rivers, and reservoirs are more susceptible to contamination than groundwater resources^11,12^. In limnological terms, reservoirs are considered artificial lakes, and many types of stagnant water bodies, including small ponds and reservoirs, can be classified as lakes depending on their size and hydrological characteristics^13^. Nowadays, the role of dams has expanded, and their importance now extends beyond ensuring and regulating water quantity to include water quality control as well^14,15^. Natural and climatic characteristics of the watershed, such as rainfall type, intensity, sediment production, and pollution from human activities like agriculture, exploitation, wastewater discharge, and other pollutants along the river, all impact reservoir water quality^16,17^. Additionally, dam construction and surface water storage can lead to changes in physical, chemical, and biological water properties due to factors like evaporation, stagnant water, thermal stratification in the reservoir, sedimentation, nutrient enrichment, and chemical changes in lake water^18,19^. Therefore, determining the water quality status is essential for implementing effective strategies to prevent water quality decline or improve it^20^. Recently, advanced data analysis approaches have been developed to simulate and predict changes in water quality. These models help analyze water quality trends over time and identify critical points requiring management intervention^21^. Statistical techniques like trend analysis, change point tests, and multivariate modeling are applied in assessing and predicting water quality changes and understanding environmental and human impacts^22^.Trend and multivariate statistical analyse provide vital information to water resource managers to implement timely control and preventive measures^23,24^, which aligns with recognized management frameworks for reservoir water quality monitoring and pollution control. Recent studies have also emphasized the crucial role of riparian zone assessment and management using geospatial technologies to mitigate pollution and sustain water quality in reservoir and river systems^25^.

### Literature review

#### Spatiotemporal trends

Kannel et al.^26^ evaluated the spatiotemporal variations in the water quality of the Bagmati River in Nepal by examining 23 parameters over four seasons. The results indicated that water quality in rural areas was affected by human sewage, while urban areas were severely polluted with untreated sewage. Statistical analyses identified three distinct pollution groups, and pollution sources were identified using statistical techniques to evaluate water quality. Shuhaimi-Othman et al.^27^ investigated water quality variations in Lake Chini, Malaysia. The results showed that the water quality of the lake was categorized as class II, suitable for recreational activities. Various parameters, such as DO, pH, TSS, and chlorophyll-a, exhibited the most significant spatiotemporal variations. The study confirmed the mesotrophic status of the lake for chlorophyll-a and indicated that water quality was influenced by various environmental factors. Randhir & Yurtseven^28^ assessed the spatiotemporal variations in irrigation water quality in the Uluabat Lake Basin, Turkey. The study used clustering, discriminant, principal component, and factor analyses to show that parameters like salinity, sodium, boron, and alkalinity had substantial impacts on irrigation water quality. The results emphasized the importance of proper water management to prevent the continuous use of low-quality water for agriculture and soil health protection. Pratama et al.^29^ analyzed the spatiotemporal trends of water quality in the Code River, Indonesia. The results showed that certain parameters, such as dissolved solids and nitrate, increased downstream, while BOD and fecal coliform reduced water quality. The study found a positive correlation between natural vegetation cover and water quality, while agricultural land and urban areas were more susceptible to pollution. Principal component analysis identified organic matter, metals, and domestic wastewater as the major contributors to water quality changes. Roy & Majumder^30^ assessed the trends in water quality changes in Rudrasagar Lake, Tripura, India, and found that overall water quality was at a moderate level during the study period, with slight deterioration in summer. Parameters such as turbidity, dissolved oxygen, and organic load had significant impacts on water quality. The results stressed the need for continuous water quality monitoring to preserve the ecosystem and local economic activities.

#### Modelling techniques

Lotfi et al.^31^ introduced a new hybrid method combining the ARIMA linear model and ORELM technique for predicting wastewater quality parameters. The study found that prediction accuracy was significantly improved, with the hybrid model performing well, especially for parameters like BOD and COD. The BOD model, with an R-squared value of 0.99, provided the best prediction, demonstrating the significant improvement in wastewater quality prediction through the integration of linear and nonlinear approaches. Tian et al.^32^ developed an integrated framework for forecasting potential water pollution areas in large watersheds using spatial-temporal graph convolution networks (STGCN). This study demonstrated that the STGCN model outperformed traditional models such as RNN, LSTM, and GRU in prediction accuracy. The results proved the framework’s ability to identify high-risk pollution areas in the Yangtze River Basin and support effective watershed management and large-scale water pollution prediction. Chin^33^ analyzed water quality in Biscayne Bay using cluster analysis and correlation. The study found average water quality at some stations, with nutrients like nitrogen and phosphorus influencing water quality. It identified three main pollution sources (nutrients, surface runoff, and biochemical oxygen demand nitrogen) indicating the role of clustering and correlation in managing water quality and ecosystems.

#### Pollution sources

Akkoyunlu and Akiner^34^ examined the water quality of rivers in the Sapanca Lake Basin in Turkey using three international water quality indices. This study aimed to assess pollution levels and compare indices, leading to the development of a modified model to reduce the number of pollution parameters. The results revealed a good relationship between the modified indices and the water quality index, identifying nutrient enrichment threats to Lake Sapanca and its feeding rivers. Abd Wahab et al.^35^ evaluated the hydrological and water quality conditions in the Kenyir Lake Basin and found that sedimentation rates were increasing, posing a threat to the Kenyir Dam’s turbines. The water quality in the basin was classified as IIB, suitable for recreational activities, with water quality indicators such as dissolved solids, turbidity, BOD, COD, and salinity identified as significant factors. Feng et al.^36^ examined water pollution scenarios and policy options for reducing pollution loads in China using the Grey Water Footprint (GWF) assessment. The study found that current policies could reduce GWF by 15.0% to 39.9%, although they were less effective in eight provinces. In a scenario involving technological improvements, GWF could be reduced by 54.9% to 71.1%. The study also evaluated policy options for freshwater resource protection in China.

Water quality is affected by spatiotemporal variations in physical, chemical, and biological parameters, with statistical and multivariate analyses effectively identifying trends. Advanced modeling approaches enhance prediction accuracy and detect high-risk pollution areas, while urban, industrial, and agricultural sources are key contributors. These results indicate the need for integrated trend analysis, modeling, and pollution source management to optimize reservoir water quality. The reviewed studies have revealed the significant role of spatiotemporal variations in water quality and the identification of pollution sources using various analytical techniques. Dynamic assessment of water quality is necessary over different geographical regions and various driving forces of water pollutants, particularly in the context of pollutants entering reservoirs and dam systems. This temporal analysis provides detailed indications into critical water quality parameters and identifies potential high-risk pollution events, enabling more accurate prediction of future water quality changes and supporting proactive water resource management and pollution control strategies.

### Scope and objective

The analysis of trends and dynamics of water quality parameters is a crucial tool for assessing temporal changes and identifying long-term patterns in water resources^37,38^. Water quality assessment is crucial for understanding pollution impacts on rivers across diverse land uses and population centers^39^. In this context, statistical and graphical methods are widely used to detect meaningful trends in time series data^40,41^. Additionally, graphical representations such as box plots and violin plots provide a more detailed analysis of the distribution and variability of water quality parameters over different time scales^42^. These methods help analysts to effectively and deeply investigate changes in water parameters and identify dynamic patterns within the data. The use of probability density function is also valuable for examining the normal distribution of data and identifying anomalies, human impacts, or climatic changes in the trends of water quality. This tool enables analysts to simulate distribution patterns of water quality parameters and examine their temporal variations across different periods. As a result, these methods can effectively assess the dynamics of water quality parameters over time and reveal the various environmental and human influences on them. The Sardasht Reservoir Dam, located in the northwest of Iran in a mountainous region, has been selected for assessing the temporal trends of key physical, chemical, and biological water quality parameters. Analyzing the changes in these indicators over time is essential for understanding the effects of pollution, natural and human-induced changes, and providing management strategies to improve water quality. Given the importance of the Sardasht Dam in water supply and sustaining irrigation water needs, examining the temporal trends of water quality parameters in terms of pollution is crucial for monitoring and optimizing water resources in the region. While several studies have examined spatiotemporal water quality variations in rivers and lakes, there remains a lack of comprehensive analyses targeting reservoir dams in northwest Iran. This study addresses this gap by investigating temporal dynamics of pollutant parameters at the Sardasht Reservoir Dam, providing critical insights for regional water resource management. Integrating Mann-Kendall with box plots, violin plots, and probability density functions provides a more comprehensive view of trends, variability, and anomalies in water quality, indicating patterns that statistical tests alone might miss. This study aims to evaluate the temporal trends of pollutant parameters in the water quality entering the Sardasht Reservoir Dam in a mountainous area in northwest Iran. The research utilizes statistical methods and trend analysis models to analyze water quality changes, which will be effective in understanding the factors influencing water quality parameters, controlling pollution, and optimizing water use.

## Materials and methods

### Description of the study area

**Fig. 1:**
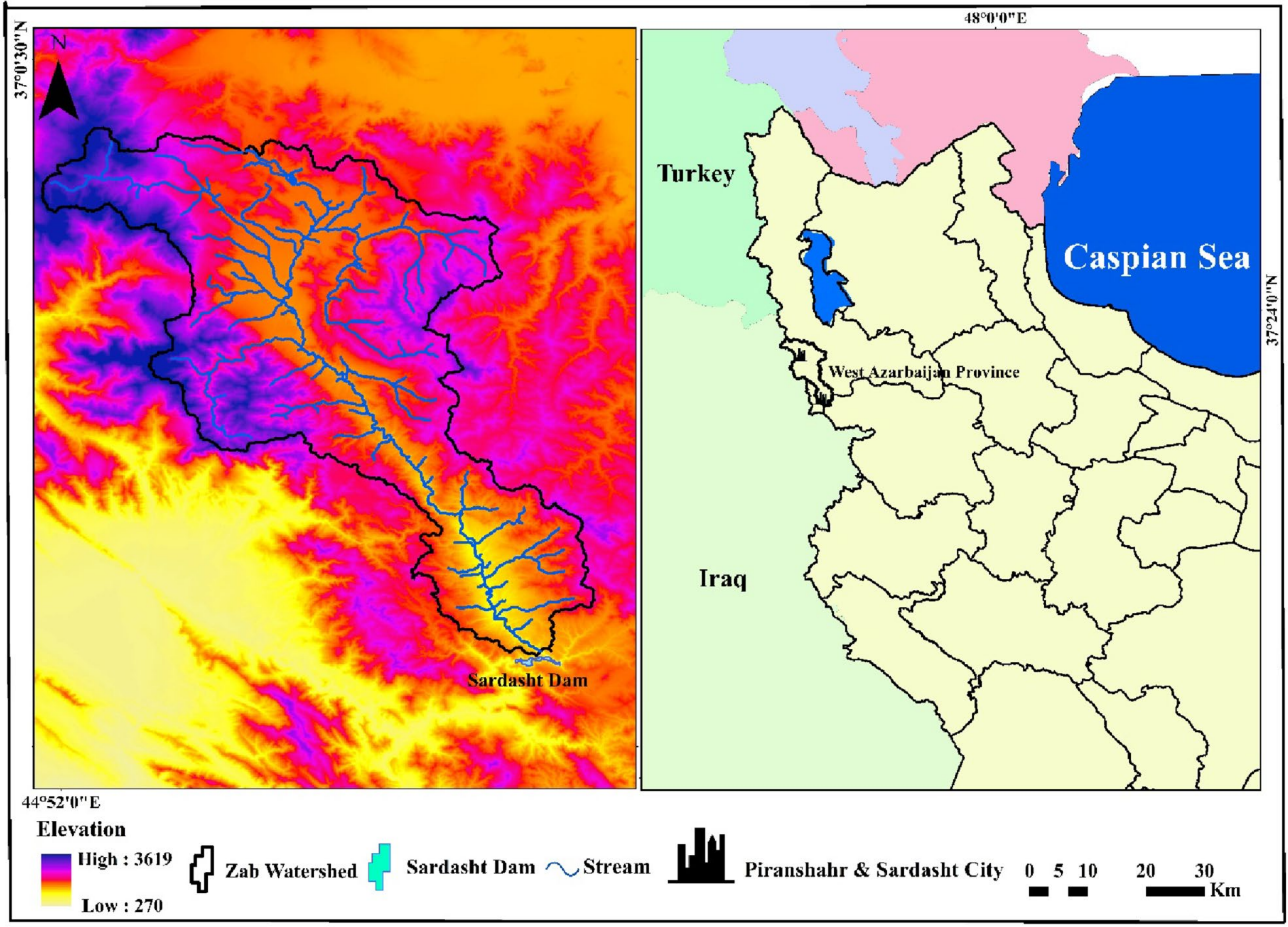
The geographic location of Sardasht Dam in West Azarbaijan and Iran (Map processing and creation were carried out by the researchers using ArcMap within ArcGIS version 10.1^46^.

The Sardasht Dam is located in the northwest of Iran, in the southwest of Sardasht County, West Azerbaijan Province. The dam is situated on the Zayandeh River, 13 kilometers southeast of Sardasht city. Its exact geographic coordinates are Latitude: 36° 32.4’ N and Longitude: 45° 58.8’ E^43^. The Sardasht Dam is an embankment dam with a clay core, with a height of 112 m and a length of 280 m at the crest. Construction of the dam began in 2009 and was completed in 2017. The Sardasht Dam has a hydroelectric power plant with a capacity of 150 MW, capable of generating 422 gigawatt-hours of electricity annually. The dam is designed with a reservoir volume of 388 million cubic meters and a normal elevation of 840 m above sea level^44^. The primary objectives of constructing the Sardasht Dam include providing drinking water, agricultural irrigation, hydropower generation, and flood control in the region. The dam plays an important role in water resource management and improving the water and electricity situation in the area^45^. Land use, vegetation cover, and precipitation were not included due to limited data availability at the required resolution. Moreover, since water quality samples were collected at different times, it was not feasible to account for the effects of land use and precipitation. The study instead focused primarily on other human-related factors influencing water quality. The geographic location of the Sardasht Dam is shown in Fig. 1.

### Water quality data set

In this study, water quality data, including important physical, chemical, and biological parameters, collected by the West Azerbaijan Province Department of Environmental Protection over a period from 2014 to 2024, were analyzed. The data used in this research include various physical, chemical, and biological water quality parameters recorded during regular monitoring years at the Sardasht Dam inflow station. The water quality parameters analyzed at the Sardasht Dam included physical, chemical, and biological indicators. Physical parameters comprised temperature (Temp, °C), electrical conductivity (EC, µS/cm), total dissolved solids (TDS, mg/L), and total suspended solids (TSS, mg/L). Chemical parameters included biochemical oxygen demand (BOD, mg/L), chemical oxygen demand (COD, mg/L), dissolved oxygen (DO, % saturation), pH (dimensionless), nitrates (NO3-, mg/L), nitrites (NO2-, mg/L), and heavy metals such as cadmium (Cd, ppm), copper (Cu, ppm), chromium (Cr, ppm), lead (Pb, ppm), iron (Fe, ppm), manganese (Mn, mg/L), and zinc (Zn, ppm). The biological parameter considered was total coliforms (TC, Most Probable Number per 100 mL). Other measured parameters were not included in the analysis due to insufficient sampling frequency. These parameters provided a comprehensive assessment of water quality entering the reservoir.

### Research methodology

In this phase, the Mann-Kendall test and Sen’s slope were applied to determine the trends of water quality parameters^47–49^. Although seasonal variations can influence water quality parameters, the sampling frequency and temporal distribution of data in this study were irregular, which limits the applicability of seasonal trend analysis methods.

To quantitatively illustrate the irregular temporal distribution of sampling, the dates of all collected samples were converted from the Solar Hijri calendar to the Gregorian calendar. The monthly and seasonal frequency of sampling is summarized in Table 1.

**Table 1:** Summary of sampling frequency by month and season at the Sardasht Dam inflow station (2014–2024).

Month	Sample Count	Season	Seasonal Count	Seasonal %
January	2	Winter	4	9.1%
February	0			
March	0			
April	4	Spring	12	27.3%
May	6			
June	6		14	31.8%
July	5	Summer		
August	3			
September	4		14	31.8%
October	7	Autumn		
November	0			
December	3			
Total	44		44	100%

As shown, sampling was highly irregular and seasonally biased. For instance, nearly 70% of all samples were collected during Spring and Summer (March to August), while the Winter season (December to February) is severely underrepresented, comprising less than 10% of samples. Specific months such as March and November had no samples at all. This irregular and unbalanced sampling frequency fundamentally limits the applicability of seasonal trend analysis methods, such as the Seasonal Mann-Kendall test. Consequently, it becomes challenging to definitively separate strong seasonal signals (such as the flushing of nitrates (NO₃⁻) following spring rainfall and fertilizer application) from baseline anthropogenic impacts. Furthermore, the infrequent and clustered sampling likely fails to capture the full magnitude, duration, and frequency of episodic pollution events (e.g., spikes in COD or Pb). Therefore, the long-term monotonic trends identified in this study should be interpreted with the understanding that finer temporal-scale dynamics, including true seasonal patterns and episodic events, require future investigation with systematic, high-frequency monitoring.

Therefore, Mann-Kendall and Sen’s slope tests were selected as robust non-parametric methods suitable for detecting monotonic trends over the entire study period^50,51^. These methods provide reliable assessments of long-term trends without assuming seasonal periodicity, allowing us to focus on overall temporal dynamics and anthropogenic impacts on water quality. Future studies with more frequent and evenly distributed seasonal data could incorporate seasonal Mann-Kendall tests to evaluate intra-annual variations. Given that this study evaluates temporal trends across multiple water quality parameters (*n* = 18), the interpretation of p-values is framed within an exploratory trend-analysis context. Accordingly, greater emphasis is placed on the direction and consistency of detected trends rather than on strict threshold-based significance alone, particularly for p-values close to 0.05. The reported p-values therefore provide a robust insights of temporal behavior, while values close to the 0.05 threshold are interpreted with appropriate caution.

**Fig. 2:**
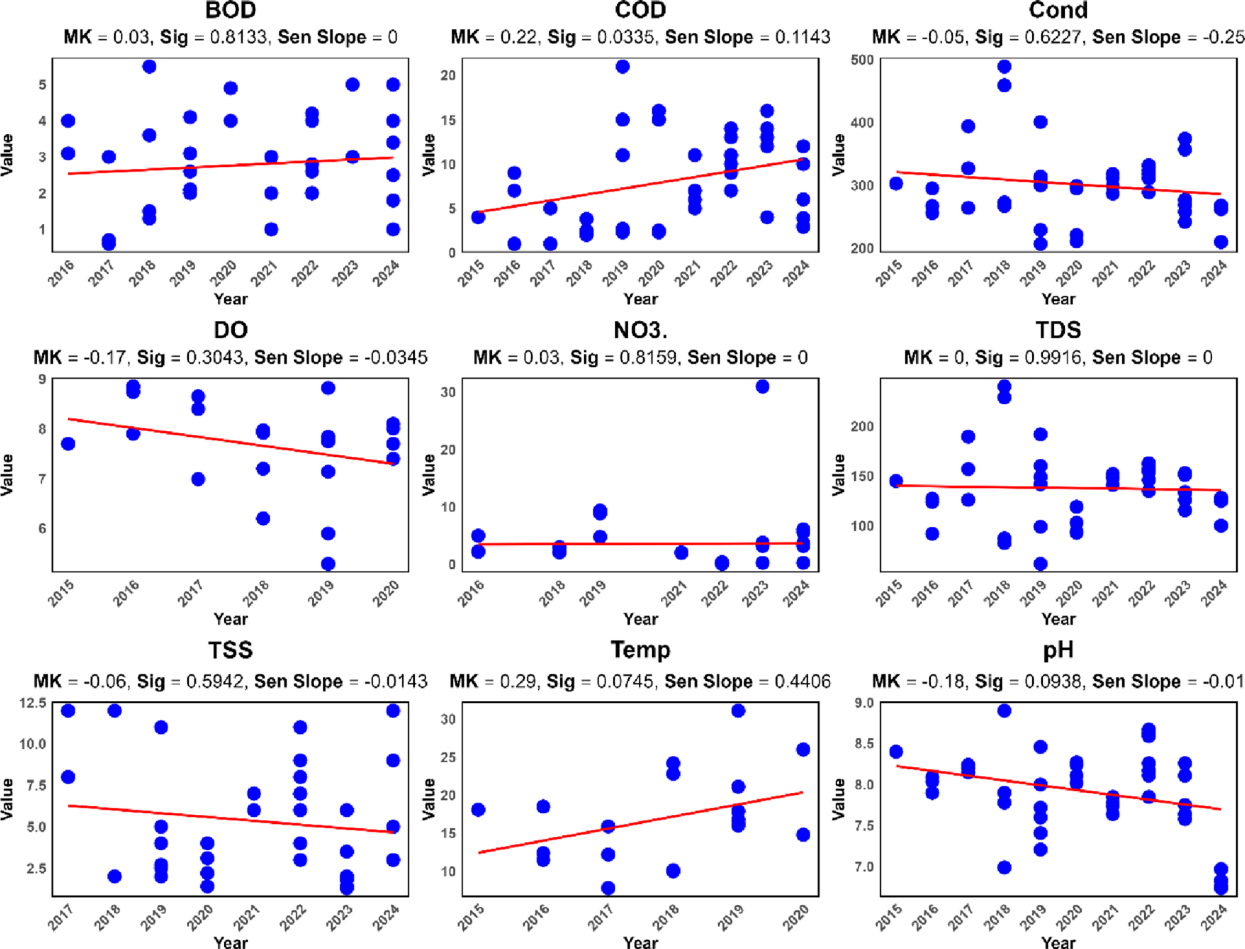
Temporal trends of water quality parameters at the station located at the inlet of Sardasht Dam.

**Fig. 3:**
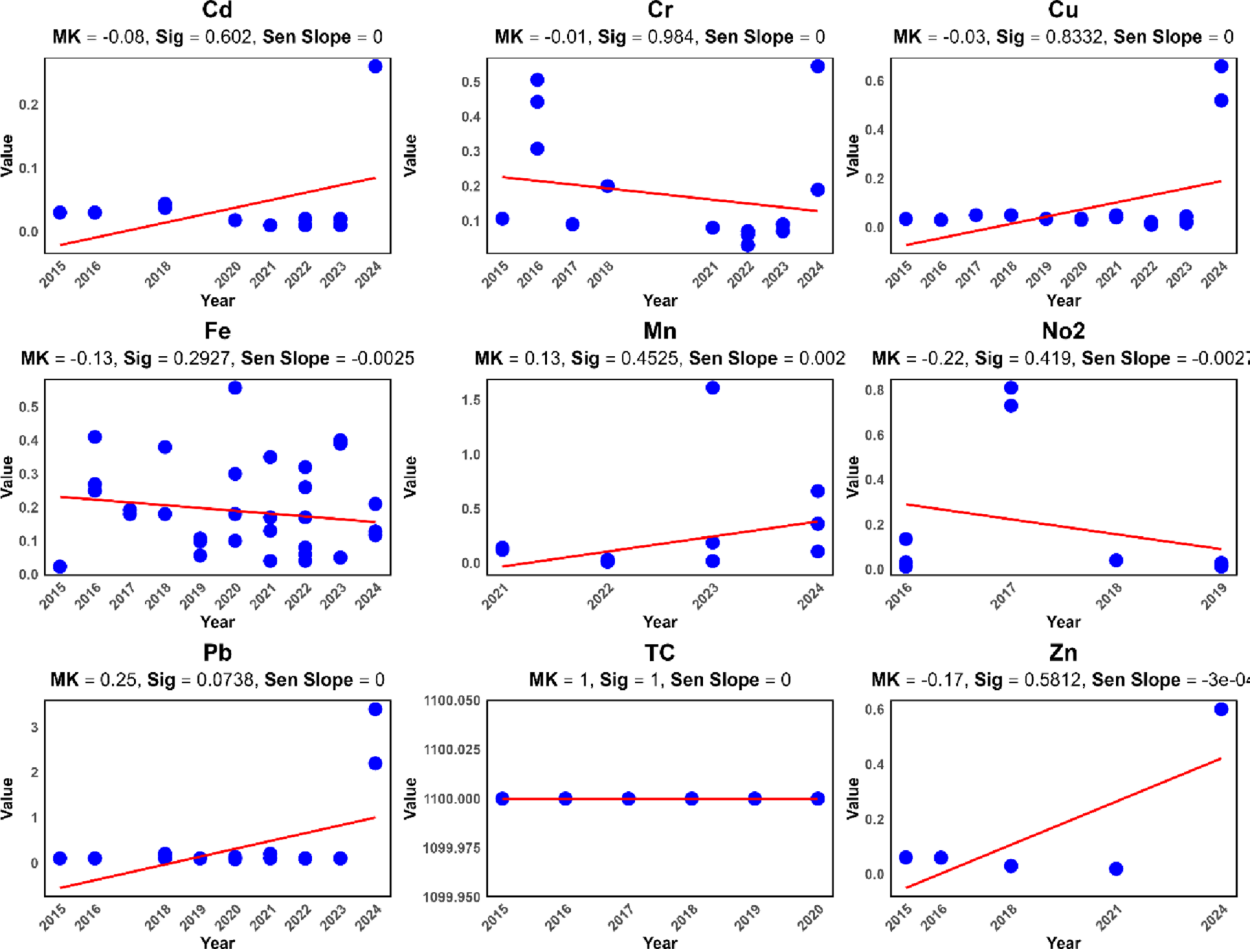
Temporal trends of water quality parameters at the station located at the inlet of Sardasht Dam.

Additionally, trend lines were added to the charts using a linear regression model. Linear regression trend lines were added to the time-series charts (Figs. 2 and 3) solely for visual aid and to provide a preliminary, intuitive depiction of the overall direction of change over time. The primary and statistically robust quantification of trends was performed using the non-parametric Mann-Kendall test and Sen’s slope estimator, the results of which are reported in Table 3.

Box plots were also generated for all water quality parameters on a logarithmic scale^52^. Further analysis was conducted using violin plots to assess the distribution of water quality parameter values. Furthermore, the probability density function (PDF) was employed to explain the density and normal distribution of water quality parameters, as well as to calculate the mean and standard deviation^53^. The PDF is used to evaluate data distributions, indicating the likelihood of different values of water quality parameters occurring within specific ranges^54^. It helps in identifying data distribution patterns, comparing distributions of various sampling parameters, and assessing temporal changes in water quality. This tool also assists in detecting anomalies or extreme values, and by comparing data with standard distributions (e.g., normal distribution), it can be used to analyze trends in water quality changes and determine the impact of human activities or climate changes on water resources^55^. In this study, R software was used along with the tidyverse package for data manipulation and visualization^56^. The zoo package was utilized for the na.approx function, and the Kendall package was employed for the Mann-Kendall test^57,58^. Additionally, ggplot2 was used for plotting, and patchwork was employed for combining the plots^59^.

## Results and discussion

The Table 2 provides descriptive statistics for various water quality parameters at Sardasht Dam.

**Table 2:** Descriptive statistics of water quality parameters used in the analysis of Sardasht Dam.

Parameter	Mean	Std. Error	Median	Mode	STDEV	Sample Var.	Range	Min	Max	Kurtosis	Skewness
TSS	5.3	0.6	4.0	3.0	3.4	11.5	10.7	1.3	12.0	−0.6	0.7
NO3-	3.6	1.1	2.3	0.1	5.8	33.1	30.9	0.1	31.0	18.6	4.0
pH	7.9	0.1	8.0	8.1	0.5	0.3	2.2	6.7	8.9	0.2	−0.6
Temp	17.4	1.3	16.3	14.8	6.1	36.6	23.3	7.8	31.1	−0.1	0.6
EC	300.1	9.0	299.0	311.0	59.1	3487.0	282.0	207.0	489.0	2.3	1.2
TDS	137.7	5.4	141.0	126.0	35.3	1244.9	178.3	61.7	240.0	1.4	0.6
BOD	2.8	0.2	2.8	2.0	1.3	1.7	4.9	0.6	5.5	−0.7	0.2
COD	8.2	0.8	7.0	11.0	5.3	27.7	20.0	1.0	21.0	−1.0	0.3
DO	7.6	0.2	7.8	7.7	0.9	0.9	3.6	5.3	8.9	0.8	−1.0
Cd	0.1	0.0	0.0	0.0	0.1	0.0	0.3	0.0	0.3	3.2	2.2
Cu	0.1	0.0	0.0	0.1	0.2	0.0	0.7	0.0	0.7	5.8	2.7
Cr	0.2	0.0	0.1	0.1	0.2	0.0	0.5	0.0	0.5	1.5	1.7
Pb	0.4	0.2	0.1	0.1	0.9	0.8	3.3	0.1	3.4	6.0	2.7
Fe	0.2	0.0	0.2	0.2	0.1	0.0	0.5	0.0	0.6	0.2	0.9
No2	0.2	0.1	0.0	0.0	0.3	0.1	0.8	0.0	0.8	1.4	1.7
Zn	0.2	0.1	0.1	0.1	0.2	0.1	0.6	0.0	0.6	1.4	1.8
TC	1100	0	1100	1100	0	0	0	1100	1100	na	na
Mn	0.2	0.1	0.0	0.0	0.4	0.1	1.6	0.0	1.6	11.7	3.3

According to Table 2, Nitrate (NO3-) shows a significant variation with a range of 30.9 mg/L (min: 0.1 mg/L, max: 31.0 mg/L), indicating high fluctuations. In comparison, TSS has a smaller range of 10.7 mg/L (min: 1.3 mg/L, max: 12.0 mg/L), signifying more stable water quality regarding suspended solids. EC and TDS exhibit considerable variability with standard deviations of 59.1 µS/cm and 35.3 mg/L, respectively. pH levels vary moderately, with a range of 2.2 (min: 6.7, max: 8.9), while Dissolved Oxygen (DO) remains relatively stable, with a range of only 3.6%. Heavy metals such as Cadmium (Cd), Copper (Cu), and Chromium (Cr) show low concentrations with small standard deviations, but Lead (Pb) has a range of 3.3 ppm (min: 0.1 ppm, max: 3.4 ppm). The Temperature (Temp) shows a wide range of 23.3 °C (min: 7.8 °C, max: 31.1 °C), indicating significant fluctuations in water temperature. BOD and COD also show notable variations, with COD having a range of 20.0 mg/L (min: 1.0 mg/L, max: 21.0 mg/L). Significant variations are observed across several parameters, particularly Nitrate (NO3-), Electrical Conductivity (EC), Lead (Pb), and Temperature (Temp). These fluctuations indicate the need for regular water quality monitoring. The substantial differences in nutrient levels and heavy metals, such as Pb, ensures the safe level of water quality at the Sardasht dam.

The temporal trends of water quality parameters at the station located at the inlet of Sardasht Dam are shown in Figs. 2 and 3.

The Table 3 presents the trends in water quality parameters at Sardasht Dam station, indicating their changes over time based on Mann-Kendall, and Sen’s Slope.

**Table 3:** Numerical trend of changes in water quality parameters over time at the Sardasht Dam station.

WQ parameter	Tau	p_value	Sen_Slope	Trend_Direction	Significance
BOD	0.029	0.813	0.000	Decreasing	-
COD	0.224	0.033	0.114	Increasing	*
EC	−0.053	0.623	−0.250	Decreasing	-
DO	−0.167	0.304	−0.035	Decreasing	-
NO3.	0.033	0.816	0.000	Decreasing	-
TDS	0.002	0.992	0.000	Decreasing	-
TSS	−0.065	0.594	−0.014	Decreasing	-
Temp	0.287	0.075	0.441	Increasing	†
pH	−0.179	0.094	−0.010	Decreasing	†
Cd	−0.075	0.602	0.000	Decreasing	-
Cr	−0.006	0.984	0.000	Decreasing	-
Cu	−0.027	0.833	0.000	Decreasing	-
Fe	−0.127	0.293	−0.003	Decreasing	-
Mn	0.130	0.453	0.002	Increasing	-
No2	−0.225	0.419	−0.003	Decreasing	-
Pb	0.252	0.074	0.000	Decreasing	†
TC	1.000	1.000	0.000	Decreasing	-
Zn	−0.167	0.581	0.000	Decreasing	-

* denotes *p* < 0.05, and † denotes marginal significance (*p* < 0.10).

Table 3 shows that most parameters, including BOD (Tau = 0.029, *p* = 0.813), TDS (*p* = 0.992), NO3- (*p* = 0.816), and pH (*p* = 0.094), exhibit slight decreasing trends, though many are not statistically significant. COD (Tau = 0.224, *p* = 0.033) shows a significant increase, while Temp (Tau = 0.287, *p* = 0.075) and Mn (Tau = 0.130, *p* = 0.453) also trend upward, possibly indicating warming effects or pollution inputs. While parameters such as Temp (*p* = 0.075), pH (*p* = 0.094), and Pb (*p* = 0.074) exhibit trends of potential interest (marked with †), their p-values exceed the conventional 0.05 threshold for statistical significance. Therefore, these trends should be interpreted as preliminary suggestions that warrant further investigation rather than conclusive evidence of change. Caution is advised in drawing strong management conclusions based solely on these marginally significant results.

The significance of the increasing COD trend (*p* = 0.033) should be interpreted in the context of multiple comparisons across 18 parameters. The Sen’s slope estimator for COD is 0.114 mg/L per year. Over the 10-year study period, this corresponds to an estimated increase of approximately 1.14 mg/L. While statistically discernible, the practical or environmental significance of this rate of change should be evaluated in relation to local water quality standards and the reservoir’s assimilation capacity. This gradual increase may indicate a growing background load of organic pollutants, warranting continued monitoring to prevent future deterioration.

The constant TC value (1100 MPN/100 mL) results from the laboratory’s reporting protocol, which records any concentration at or above this threshold as 1100. This is not due to insufficient data. The data confirm chronic fecal contamination but lack the variance needed for statistical analysis.

Electrical Conductivity (EC, *p* = 0.623) and DO (*p* = 0.304) show minor declines over time. The COD increase, with its significant p-value, suggests that organic pollution may be intensifying in the water body.

The grouping of parameters based on trends shows a broad similarity in direction, with most parameters (e.g., TSS, Cd, Cr, Cu, Fe, and Zn) displaying decreasing trends. This clustering indicates a collective reduction in specific water quality attributes. However, the increasing trends of COD, Temp, and Mn form a smaller group, potentially linked to emerging pollutants or environmental changes. The increasing trends in COD, temperature (Temp), and manganese (Mn) in water quality indicate significant changes, likely caused by various factors. Elevated COD levels may result from the inflow of organic pollutants such as urban or agricultural wastewater, showing reduced oxygen availability required for organic matter decomposition. The episodic spikes in NO3 are consistent with and could potentially be linked to patterns of fertilizer application on farmlands followed by rainfall events, which generate polluted runoff.

The interpretation of episodic spikes in parameters like NO₃⁻ (linked to agricultural runoff) and Pb (indicative of point-source discharges) must consider the irregular sampling schedule (Table 1). The clustering of samples in specific months and the under-sampling of entire seasons mean our dataset may not fully represent the frequency or peak intensity of such pollution events.

Similarly, the sporadic peaks in Pb suggest intermittent point sources, which could include leachate from unregulated solid waste dumps observed near the river and/or contamination from local small-scale industrial activities. In the specific context of the Sardasht Dam watershed, the most significant sources of this organic load are likely the direct discharge of untreated domestic wastewater from riverside villages (e.g., Shivashan, Brisoo) documented in this study, combined with runoff from agricultural lands and livestock operations.

Rising temperatures could be influenced by climatic factors or warming surface waters, potentially decreasing dissolved oxygen and accelerating algae growth. Locally, this trend may be exacerbated by a reduction in riparian vegetation cover, which decreases shading, and by the input of warmer water from untreated urban discharges or agricultural return flows. Additionally, increased manganese levels might stem from industrial activities or leaching from metal-rich soils, posing health risks to consumers. Given the land use in the catchment, the increasing Mn trend is more likely linked to natural leaching from geological formations and soils, a process that can be intensified by soil erosion from deforested or steep agricultural lands, as well as from improper disposal of solid waste along the riverbanks. Trends in Sardasht Dam water quality reflect local pollution sources. Rising COD (Tau = 0.224, *p* = 0.033) is linked to untreated domestic wastewater and agricultural runoff. Increases in temperature (Tau = 0.287, *p* = 0.075) result from reduced vegetation and urban effluents, while elevated Mn (Tau = 0.130, *p* = 0.453) and Pb levels stem from soil leaching, industrial activity, and improper waste disposal. NO3- spikes are likely associated with sources such as fertilizers and septic leakage. Human activities and land use changes are key drivers of these water quality changes. These changes can have adverse effects on water quality. High COD levels reduce water usability for drinking and agricultural purposes. Elevated temperatures may foster the growth of harmful organisms and further deplete dissolved oxygen. Moreover, excessive manganese can negatively impact human health and ecosystems, especially in drinking water systems. These data were collected from a location feeding into the Sardasht Dam reservoir, directly affecting its water quality. The accumulation of organic matter (COD) in the dam can lead to problems like eutrophication and algal blooms. Higher water temperatures in the reservoir could increase evaporation rates, reducing the volume of usable water. Furthermore, elevated manganese levels may result in hazardous sediment buildup over time, increasing water treatment costs. Proper management of these inflows is crucial to maintain the dam’s water quality. The statistically significant upward trend in COD and the increase in temperature merit particular attention, as they may reflect ongoing environmental pollutants. Trends in Sardasht Dam water quality reflect local pollution sources. Rising COD and NO3- are linked to untreated domestic wastewater, septic leakage, and agricultural runoff. Increases in temperature and elevated Mn and Pb levels result from reduced vegetation, urban effluents, soil leaching, industrial activities, and improper waste disposal. Human activities and land-use changes are key drivers, proving the need for improved wastewater treatment and sustainable farming practices.

**Fig. 4:**
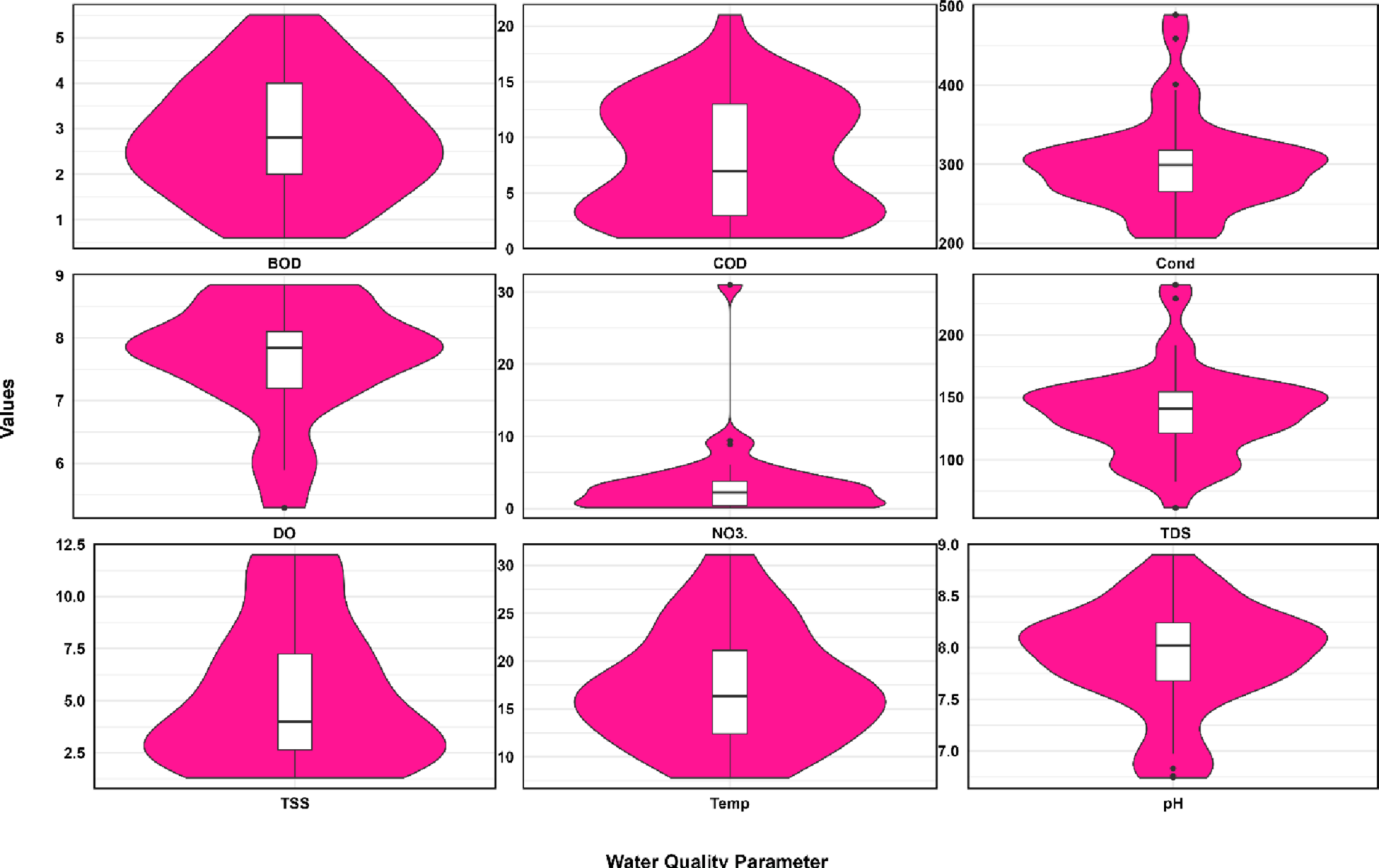
Comparison of violin plot showing changes in water quality values at the Sardasht Dam station.

**Fig. 5:**
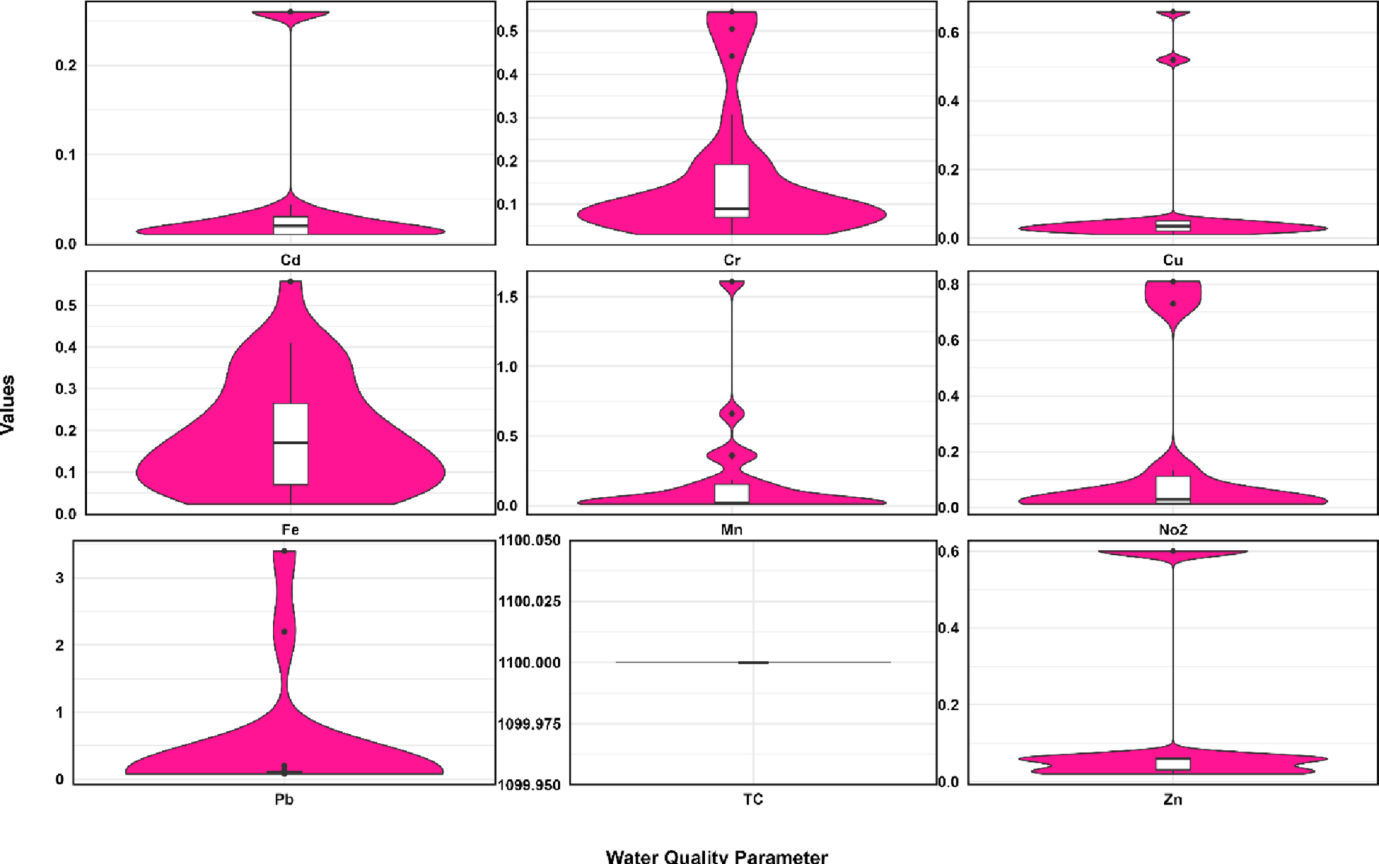
Comparison of violin plot showing changes in water quality values at the Sardasht Dam station.

The violin plots showing changes in water quality values at the Sardasht Dam station are presented in Figs. 4 and 5.

The violin plot statistics for water quality parameters at the Sardasht Dam station (Figs. 4 and 5) reveal diverse distributions. COD exhibits a significant difference between its mean (8.20) and median (7.00) with a relatively high standard deviation (5.27), proposing a skewed distribution and variability in organic pollutant levels. Similarly, NO3 shows a large disparity between its mean (3.60) and median (2.25), along with a high standard deviation (5.75), indicating irregular nitrate concentrations. Among the metals, Mn displays remarkable differences, with a mean (0.19) significantly higher than its median (0.02) and a high standard deviation (0.37). This suggests infrequent but substantial peaks in manganese levels. Pb also demonstrates considerable variability, with a mean (0.44), a much lower median (0.10), and a high standard deviation (0.92), indicating occasional spikes in lead concentrations. In contrast, Cd, Cr, and Cu show lower variability, with smaller standard deviations and closer mean-median values. Some parameters display relatively uniform distributions. DO has a small standard deviation (0.95), with its mean (7.64) and median (7.84) closely aligned, indicating stable dissolved oxygen levels. Similarly, TDS exhibits consistent values, with a mean (137.70), median (141.00), and a standard deviation (35.28), reflecting limited variation in total dissolved solids. pH, with a mean of 7.91 and a median of 8.02, has a minimal standard deviation (0.52), showing stable water acidity. The considerable differences in statistics for parameters like COD, NO3, Mn, and Pb indicate significant variability and possible episodic pollution events at the Sardasht Dam station. Conversely, more stable parameters like DO, TDS, and pH indicate relatively consistent water quality in those aspects.

**Fig. 6:**
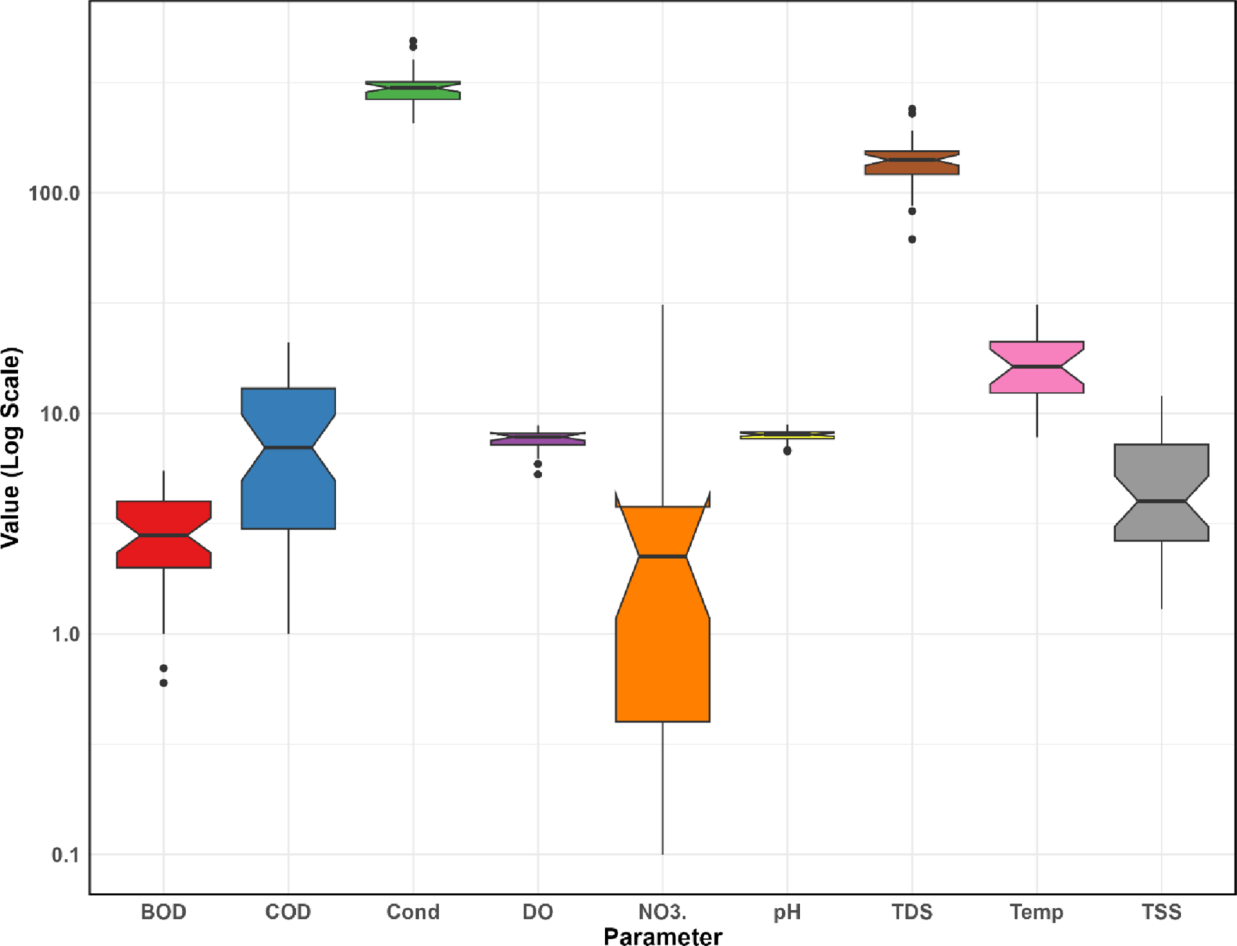
Comparison of box plot changes in water quality values at the station inlet to Sardasht Dam.

**Fig. 7:**
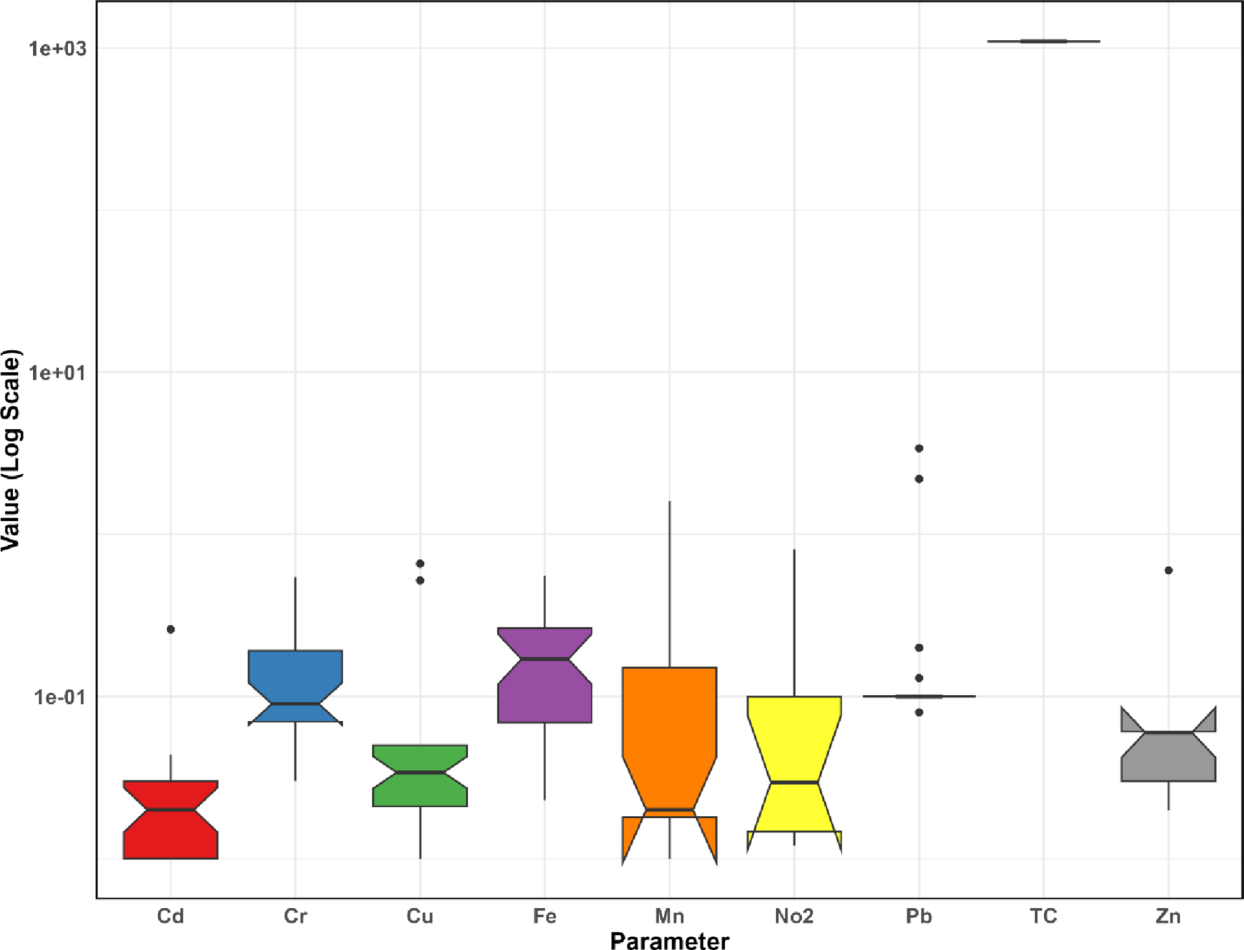
Comparison of box plot changes in water quality values at the station inlet to Sardasht Dam.

The box plot of changes in water quality values at the station inlet to Sardasht Dam is shown in Figs. 6 and 7.

The boxplot statistics for water quality at Sardasht Dam (Figs. 6 and 7) show considerable variation in some parameters. COD (max 21.00, median 7.00) and NO3 (max 31.00, median 2.25) indicate episodic peaks, while Mn (max 1.61, median 0.02) and Pb (max 3.40, median 0.10) show occasional spikes. In contrast, Cd, Cr, DO (median 7.84, range 5.29–8.85), pH (median 8.02, range 6.74–8.90), and TDS (median 141.00, max 240.00) are more stable, indicating consistent water quality in these aspects.

**Fig. 8:**
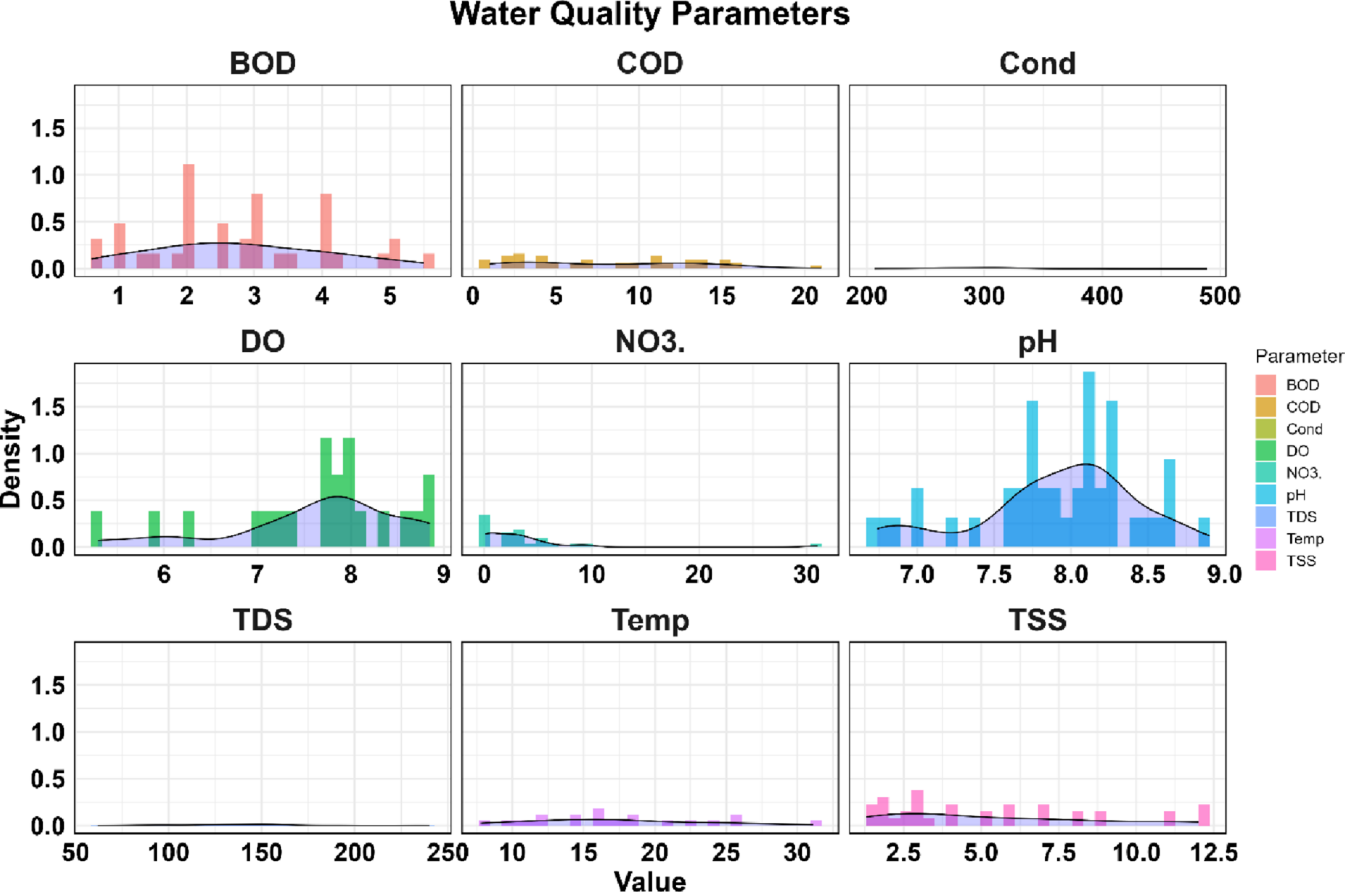
Comparison of Probability Density Function (PDF) plots of water quality values at the station inlet to Sardasht Dam.

**Fig. 9:**
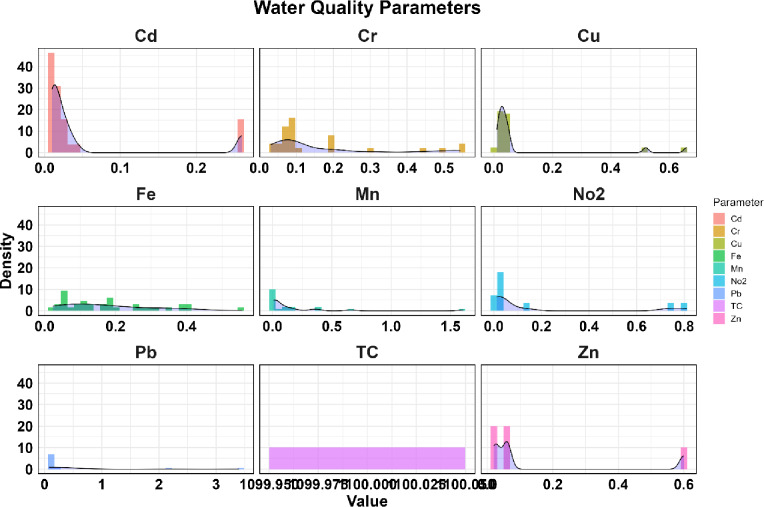
Comparison of Probability Density Function (PDF) plots of water quality values at the station inlet to Sardasht Dam.

The PDF plots of water quality values at the station inlet to Sardasht Dam are shown in Figs. 8 and 9.

Figures 8 and 9 show that COD, NO3, and Pb have large differences between mean and median values and high standard deviations, indicating episodic fluctuations and potential contamination events (COD mean 8.20, SD 5.27; NO3 mean 3.60, SD 5.75; Pb mean 0.44, median 0.10, SD 0.92). In contrast, BOD, DO, and pH are more stable (BOD mean 2.79, SD 1.29; pH mean 7.91, SD 0.52), showing consistent water quality. Trace metals like Mn (mean 0.19, SD 0.37) and Cu (mean 0.09, SD 0.18) show some variability, showing occasional risk. Overall, the PDFs indicate fluctuating patterns for NO3 and Pb, while pH and DO remain consistent.

The PDF analysis provided critical, complementary insights beyond central tendency statistics. For parameters like COD, NO₃, and Pb, the PDF plots visually confirm their non-normal, right-skewed distributions, characterized by a long tail towards higher values. This shape quantifies the likelihood and magnitude of extreme pollution events. For instance, the pronounced right tail in Pb’s PDF (Fig. 9) objectively demonstrates that while most measurements are low (near the median of 0.10 mg/L), there is a measurable probability of encountering dangerously high concentrations (exceeding 3 mg/L), aligning with the episodic spikes identified in boxplots and time-series. Conversely, the near-normal, symmetric PDFs of pH and DO (Fig. 8) visually corroborate their statistical stability and lower vulnerability to extreme deviations. Thus, the PDFs were instrumental in transitioning from describing ‘variability’ to probabilistically characterizing the ‘risk of exceedance’ for key pollutants, offering a more nuanced understanding of the threat profile entering the reservoir.

The results of the water quality changes and pollution assessment in the study area align with previous studies that indicate fluctuations in water quality due to agricultural, industrial, and domestic wastewater pollution. Significant changes in parameters such as NO3- and COD were observed, clearly influenced by agricultural and urban pollution, consistent Pratama et al^29^., who analyzed the relationship between vegetation cover and water quality. The observed fluctuations in NO3- at Sardasht Dam, especially during rainy seasons and agricultural runoff, indicate significant seasonal variability. Similar trends were reported by Majumdar & Avishek^62^ in the Danro River, where nitrate levels and pollution indices differed markedly between monsoon and non-monsoon periods, which demonstrates the importance of considering seasonal effects in water quality assessments to manage nutrient loads and anticipate episodic pollution events.

Additionally, the present study recorded water quality degradation caused by urban and rural wastewater inflows, attributed to agricultural and human activities, in line with Kannel et al^26^., who reported significant impacts of urban wastewater on COD and NO3- in areas with industrial and human activities. Similarly, long-term monitoring of river water quality has shown consistent exceedances of BOD and COD limits along with high coliform counts, highlighting the persistent nature of organic and biological pollution in aquatic systems^61^. At Sardasht Dam, these parameters showed less variation, indicating relative water quality stability, similar to Shuhaimi-Othman et al^27^., who noted stability in parameters such as pH and DO. Furthermore, changes in heavy metal concentrations, including lead (Pb) and manganese (Mn), observed in this study are consistent with Randhir & Yurtseven^28^, who reported the effects of local pollution sources on water quality.

**Fig. 10:**
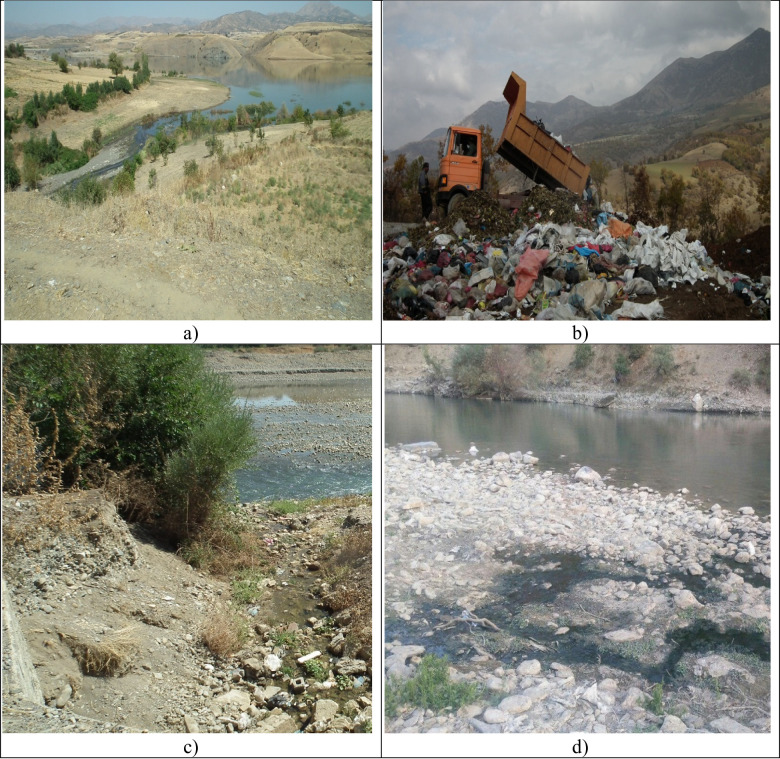
Representative upstream pollution sources affecting Sardasht Dam water quality: (**a**) wastewater discharge from Shivashan village, (**b**) waste disposal from Mirabad city, (**c**) wastewater inflow from Nalas village, (**d**) wastewater inlet from Cheko village, Mirabad protected area. These sources contribute to episodic increases in COD, NO3, and Pb concentrations in the reservoir. (All photographs were taken by the authors during field visits to the study area in 2024.).

Some images of pollution sources inflowing into the reservoir dam of the study area are shown in Fig. 10.

The detailed pollution sources and management recommendations for the study area are presented in Table 4.

**Table 4:** Pollution sources and wastewater management recommendations for Sardasht reservoir dam.

Row	Pollution Source and Origin	Type of Wastewater	Nature of Pollutant	Wastewater Discharge Type	Impact/Recommendations/Solutions	Responsible Authority
1	Improper dredging of the reservoir and presence of standing trees	Construction waste	Chemical/Biological/Physical	Non-point	Dredging and cleaning of canals and inlet to the dam reservoir to reduce phosphorus levels in water	Local Environmental Protection Department
2	Sardasht city wastewater	Domestic wastewater	Physical/Chemical	Point	- Organizing and covering the wastewater collection system for treatment plant transfer. - Developing infrastructure to collect wastewater from non-terminal points. - Separating the runoff ramp to direct rainwater to the treatment plant. - Promoting efficient drinking water use and reducing chemical consumption.	Sardasht Municipality
3	Rabat city wastewater	Domestic wastewater	Physical/Chemical	Point	Constructing a sewage treatment plant	Rabat Municipality
4	Nalas village wastewater	Domestic wastewater	Physical/Chemical	Point	Constructing a sewage treatment plant	Nalas Village Council
5	Mirabad city wastewater	Domestic wastewater	Physical/Chemical	Point	Constructing a sewage treatment plant	Mirabad Municipality
6	Slaughterhouse and waste disposal site in Sardasht city	Wastewater and leachate	Chemical	Point	- Locating a waste disposal site outside the dam’s upstream limit - Managing household waste leachate properly - Installing a sewage pipeline for the slaughterhouse and organizing the system to the treatment plant	Sardasht Municipality & Environmental Protection Dept.
7	Sand and gravel units along the Zab River	Sediments and suspended solids with high TSS	Physical	Point	- Mandating sand and gravel units to install septic tanks and sedimentation basins. - Monthly monitoring by environmental experts and certified labs	Sand & Gravel Companies
8	Fish farming units	Discharged wastewater	Chemical	Point	- Mandating fish farming units to pre-treat wastewater before discharge. - Monthly monitoring by environmental experts and certified labs	Fish Farm Owners
9	Imam Khomeini Hospital in Sardasht city	Hospital wastewater	Chemical	Point	-Mandating the health network to construct a sewage treatment plant for Imam Khomeini Hospital in Sardasht.	Health Network & Municipality
10	Villages along the Zab River leading to the dam reservoir	Wastewater	Chemical	Point	- Establishing small-scale treatment systems or pre-treatment facilities in low-population rural areas. - Installing household absorption wells for effective wastewater management.	Rural Development Office
11	Farms and orchards along the Zab River	Wastewater	Chemical	Non-point	- Encouraging farmers to use pesticides and fertilizers properly. - Banning pesticide use in orchards within a 10 km radius of the dam reservoir.	Agriculture Dept.
12	Livestock and poultry	Dead carcasses	Physical	Non-point	Monitoring livestock and poultry units to prevent disposal and burial of dead carcasses near the reservoir.	Veterinary Services
13	Rural waste	Waste and garbage	Physical	Non-point	Implementing proper waste disposal measures in rural areas.	Rural Development & Environmental Protection

The recommendations in Table 4 can be prioritized based on the severity of the associated pollutant, the immediacy of the threat to water quality, and the feasibility of implementation. High-priority actions should target sources linked to the most critical pollutants identified in this study, specifically, untreated domestic wastewater from major settlements (Sardasht, Mirabad, Nalas) contributing to rising COD and nitrate levels, and point sources linked to episodic lead (Pb) spikes. Constructing centralized sewage treatment plants for these urban areas (Rows 2, 4, 5) is therefore paramount. Medium-priority actions include controlling non-point agricultural runoff (Row 11) and managing waste from specific industrial/commercial activities like slaughterhouses and sand/gravel units (Rows 6, 7). Lower-priority initiatives, though important for long-term sustainability, involve broader institutional measures such as comprehensive rural waste management programs (Row 13). A detailed feasibility study, including cost-benefit analysis and phased implementation timelines, is recommended as the essential next step to translate these diagnostic results into an executable watershed management plan.

Recommendations are qualitatively prioritized based on the pollutant risk identified in this study. High Priority: Direct sources of COD, NO₃, and Pb from major population centers. Medium Priority: Significant non-point or regulated point sources. Low Priority: Broad institutional and long-term measures. Precise cost estimation and implementation scheduling require a subsequent feasibility study.

In the villages of Shivashan, Gerzhal Sofla, Digeh, Brisoo, and Nalas, all wastewater, including that from toilets, bathing, and other sources, is directly discharged into the river and dam reservoir, making these villages significant contributors to water pollution. Observations in upstream communities indicate that insufficient wastewater management and direct discharge of domestic effluents into the river contribute to episodic increases in nutrient and organic pollutant levels in the reservoir inflows. These practices, combined with runoff from agricultural and livestock activities, represent significant non-point sources of pollution affecting water quality at the Sardasht Dam. In other villages located upstream of the dam and along the main river leading to the reservoir, absorption wells have been constructed for toilet wastewater. However, the remaining wastewater and effluent are discharged into the river. Given the proximity of many villages to the dam reservoir or Zab River, the absorption wells often leach into the groundwater, exacerbating pollution.

During rainy seasons, the wastewater from all villages around the Zab River and upstream of the dam reservoir, along with runoff, flows into the river or the reservoir, significantly increasing the pollution load. This study focused primarily on water quality measurements at the Sardasht Dam inflow station, although runoff from rainfall events and seasonal precipitation is recognized as a contributing factor for episodic pollution peaks (particularly during rainy seasons) no direct analysis of rainfall data or river discharge patterns was performed in this study. Therefore, conclusions related to runoff are based on observed fluctuations in pollutant concentrations (e.g., COD, NO3, Pb) during periods of known precipitation events, combined with field observations of wastewater and surface runoff inflows upstream of the dam. Future studies incorporating rainfall and hydrological data are recommended to quantitatively assess the impact of runoff on reservoir water quality.

Our results on increasing COD and episodic metal pollution align with challenges documented in semi-arid and mountainous reservoirs globally, such as those in Turkey^28^ and India^62^, where agricultural runoff and inadequate wastewater treatment are key drivers. The observed warming trend in water temperature further resonates with studies attributing such changes to climatic shifts and local anthropogenic thermal inputs. This broader context underscores that the pressures on the Sardasht Dam are not isolated but part of a regional pattern in climatically sensitive zones, reinforcing the urgency of implementing integrated watershed management strategies.

Furthermore, in most villages within the study area, livestock manure and household waste have been observed to be improperly disposed of along the banks of the Zab River due to a lack of proper waste management, which significantly contributes to the water pollution burden. Effective wastewater management is essential to reduce pollutant loads entering reservoirs and to protect water quality^62^. Integrated monitoring and treatment strategies, including domestic, industrial, and agricultural sources, can significantly mitigate heavy metal and organic contamination in inflowing waters^62^. Consistent with previous studies Majumdar et al^60^; Majumdar et al^62^.; Randhir & Yurtseven^28^,, temporal analysis at Sardasht Dam shows distinct trends for heavy metals, nutrients, and physical-chemical parameters. Manganese (Mn) and lead (Pb) exhibited occasional spikes, likely from industrial activities and runoff, aligning with Majumdar et al^62^. on episodic metal contamination. A recent comprehensive risk assessment in a river basin also highlighted significant ecological and human health risks, particularly for children, due to heavy metal contamination like Cr, Cd, and Pb, underscoring the critical need for routine monitoring and treatment of water resources^63^. Similar results of significant heavy metal contamination and seasonal variability have been reported in river basin studies using pollution indices, underscoring the widespread nature of this issue^64^. NO3- and COD varied significantly over time, reflecting episodic pollution from agriculture and urban wastewater, consistent with Pratama et al^29^. and Kannel et al.^26^. Temperature, pH, TDS, and DO were relatively stable, though temperature increased slightly, potentially due to climate change, similar to trends observed in other reservoirs (Shuhaimi-Othman et al.^27^; Feng et al^36^.). These results show the need for targeted management strategies for each pollutant type to maintain water quality and ecosystem health.

### Comparison with water quality standards and risk assessment

To evaluate the potential risks associated with the observed pollutant levels, the measured concentrations of key parameters (COD, Mn, and Pb) were compared with the Iranian national standards for drinking water, ISIRI 1053^65^ and the WHO guidelines for drinking-water quality^66^. Table 5 presents the permissible limits along with the mean and maximum concentrations obtained in this study.

**Table 5:** Comparison of Key Water Quality Parameters with National and International Standards.

Parameter	Mean (this study)	Max (this study)	Iranian standard (drinking water)	WHO guideline	Risk when exceeded
COD (mg/L)	8.2	21	10	--	Ecological degradation, water treatment challenges
Mn (mg/L)	0.19	1.6	0.1	0.1	Neurological effects, aesthetic issues (staining, taste)
Pb (mg/L)	0.44	3.4	0.01	0.01	Neurodevelopmental (children), cardiovascular, renal effects

The results show that the mean COD concentration (8.2 mg/L) is below the Iranian drinking water standard (10 mg/L), although the maximum value (21 mg/L) exceeds this limit during episodic pollution events, indicating organic contamination that may cause oxygen depletion and increased treatment costs. Both the mean (0.19 mg/L) and maximum (1.6 mg/L) manganese concentrations exceed the WHO and Iranian guideline (0.1 mg/L), posing potential neurological risks and aesthetic problems. Lead presents the most serious concern, as its mean (0.44 mg/L) and maximum (3.4 mg/L) concentrations greatly surpass the permissible limit (0.01 mg/L), implying severe health risks, particularly neurodevelopmental effects in children. Intermittent peaks of Pb and Mn suggest sporadic pollution sources, such as industrial activities or improper waste disposal. In contrast, nitrate concentrations remain below the WHO guideline, indicating a lower immediate risk. Overall, Mn and Pb are identified as priority contaminants requiring urgent management in the inflow water of the Sardasht Dam.

### Implications and suggestions

The Sardasht Reservoir Dam, as a critical infrastructure in northwest Iran, plays a vital role in water resource management, requiring careful monitoring of water quality. Evaluating the temporal trends of key water quality parameters at the dam inflow station is crucial for managing pollution and optimizing water usage, particularly in a region prone to both natural and anthropogenic environmental pressures. The study’s results show significant variations in key pollutants such as COD, NO3, and manganese, which could indicate episodic contamination events or the effects of climate change. Given the dam’s multifaceted role in regional water supply, monitoring water quality parameters such as temperature, pH, and heavy metals is necessary for safeguarding public health and ecosystem stability. The increasing trend in organic pollutants and temperature could suggest a growing challenge for maintaining water quality, requiring adaptive management strategies to mitigate potential risks. The most critical pollutants identified in this study are COD, NO3, and manganese, showing significant fluctuations and increasing trends in the Sardasht Dam inflow. The highest risk areas are the inflow points from Sardasht city, Nalas village, and surrounding upstream villages along the Zab River, where untreated domestic wastewater, agricultural runoff, and livestock waste directly enter the reservoir. Heavy metal contamination, particularly Pb and Mn, although episodic, poses additional risks to water quality.

### Limitations and future research directions

A limitation of this study is that water quality analysis relies solely on data from the Sardasht Dam inflow station, which may not capture the full variability across the reservoir or downstream areas. Additionally, the study’s reliance on secondary data with potential gaps in sampling frequency for certain parameters, like heavy metals, could limit the robustness of the trend analysis and the generalizability of the results. Future research should investigate the effects of land use changes and agriculture on water quality at Sardasht Dam, focusing on COD, NO3, and manganese. Incorporating climate change models and exploring spatial variability across inflow stations will help predict trends and identify pollution sources. One limitation of this study is the lack of continuous and spatially distributed water quality data, which could improve the accuracy of trend detection and pollution assessment. A potential methodological limitation is that the Mann-Kendall test assumes no significant serial correlation. Due to irregular sampling intervals, a formal autocorrelation analysis was not conducted for all parameters. Although the test is robust, the possible influence of autocorrelation on trend significance cannot be excluded. Future studies with regular data should apply pre-whitening to address this. This study could not directly link the observed water temperature rise to climate change due to a lack of regional climatic data. The increase should be interpreted as a trend with multiple potential drivers, including local factors. Additionally, studying the ecological impacts and using advanced models like machine learning will improve monitoring and management, while long-term monitoring with predictive modeling supports sustainable water use.

## Conclusion

This study evaluates the temporal trends of pollutant parameters affecting water quality entering the Sardasht Reservoir Dam in northwest Iran. By applying statistical methods and trend analysis models, the research aims to identify factors influencing water quality, and discussion pollution source and control efforts. The study shows key water quality trends at Sardasht Dam, with a significant rise in COD indicating increased organic pollution, while most parameters like TDS and DO remain relatively stable. Higher temperatures and elevated manganese suggest additional environmental pressures. COD, NO3, Mn, and Pb show episodic pollution events, whereas DO, TDS, and pH remain consistent. These trends call for targeted pollution control measures to protect water quality. The boxplot analysis of water quality parameters at the Sardasht Dam station reveals significant variability in pollutants like COD, NO3, Mn, and Pb, indicating episodic contamination, while more stable parameters such as DO, pH, and TDS reflect consistent water quality in other aspects. The pollution sources of the Zab River and the Sardasht Dam reservoir include direct discharge of industrial, agricultural, livestock, and urban wastewater. In Nalas village, Sardasht city, and Mirabad city, the absence of proper wastewater treatment systems, improper waste disposal sites, and unauthorized sewage discharge in Sardasht have led to leachate leakage into the river and reservoir. The reduction in vegetation cover in steep lands, along with agricultural activities and increased runoff, has caused erosion and nutrient transfer (nitrates and phosphates) into the river’s inflow. Agricultural runoff which may contain pesticides and nutrients, along with pollution from sand and gravel units and inefficient wastewater treatment plants in Sardasht and Piranshahr, is considered a potential contributor to the worsening water quality. Key pollution sources affecting the Sardasht Dam reservoir include domestic wastewater from cities and villages, improper reservoir dredging, and agricultural runoff. Recommended solutions involve constructing sewage treatment plants, improving wastewater collection systems, managing industrial and agricultural discharges, and implementing monitoring programs for fish farming, livestock, and rural waste to mitigate environmental impacts.

## Data Availability

All data generated from the analyses and presented results during this study are included within this article in the form of comprehensive summary statistics and graphical representations.
